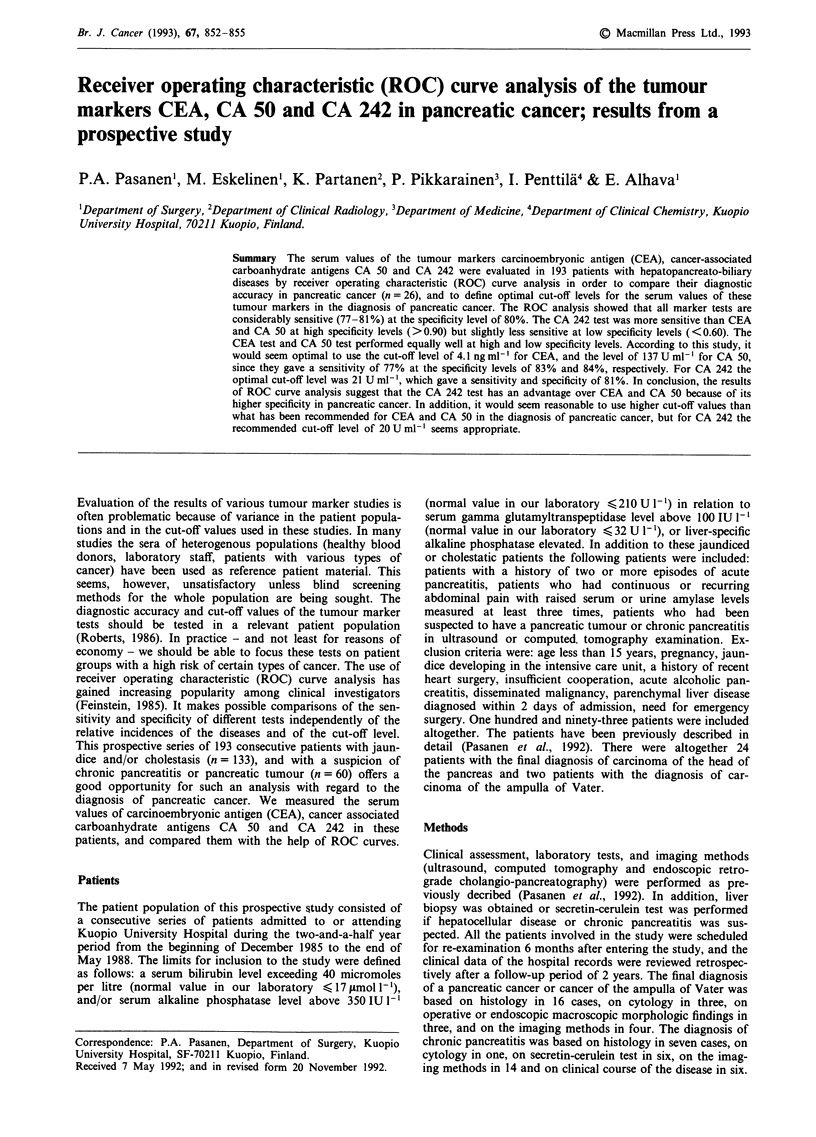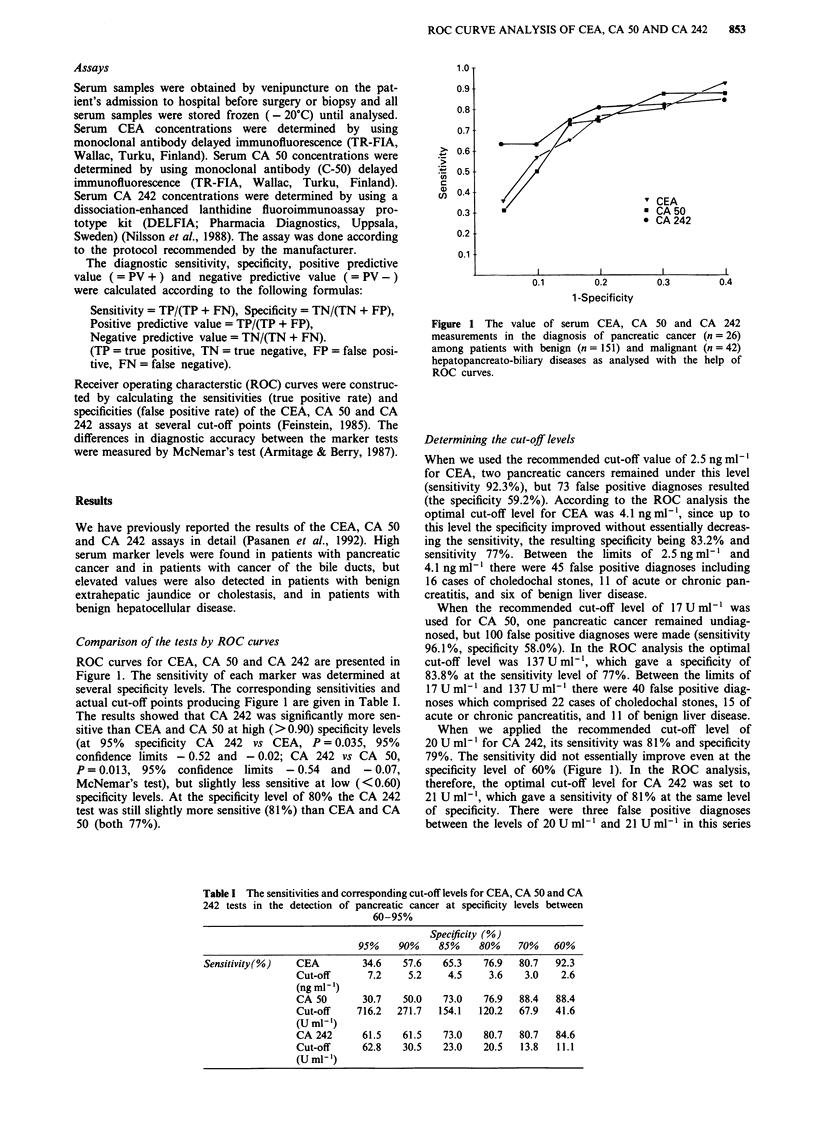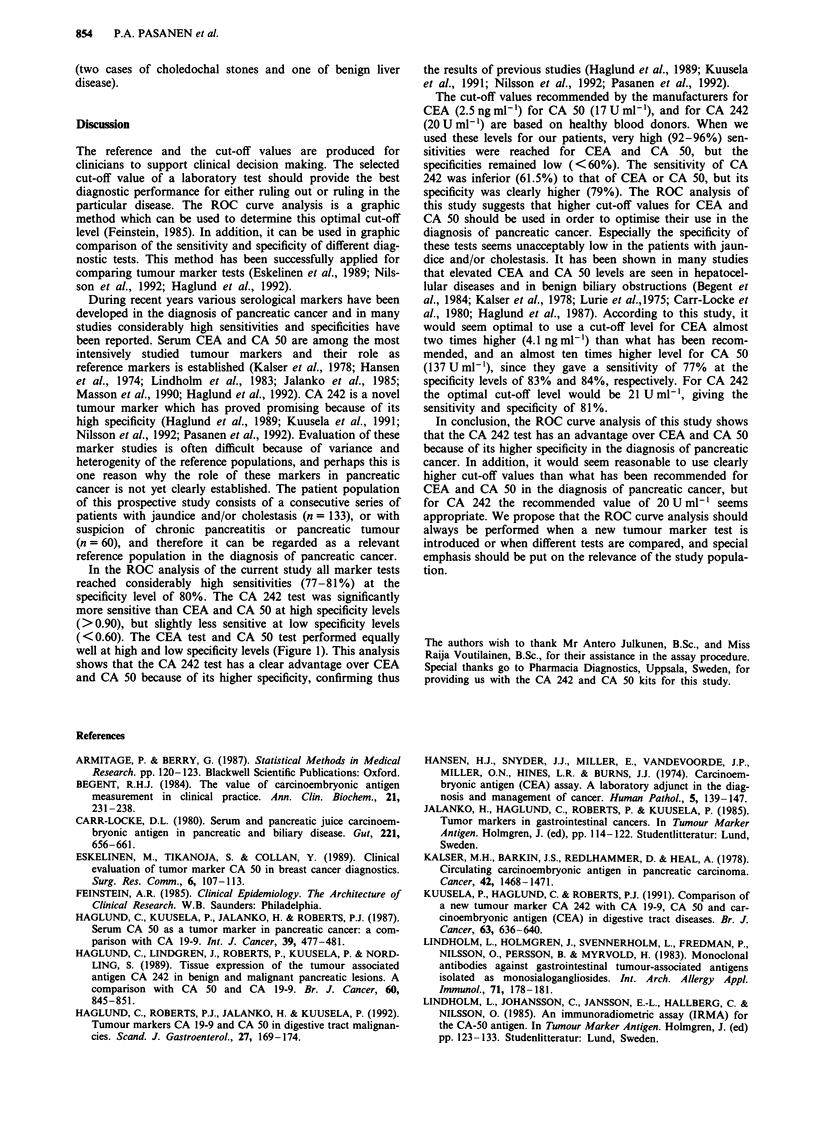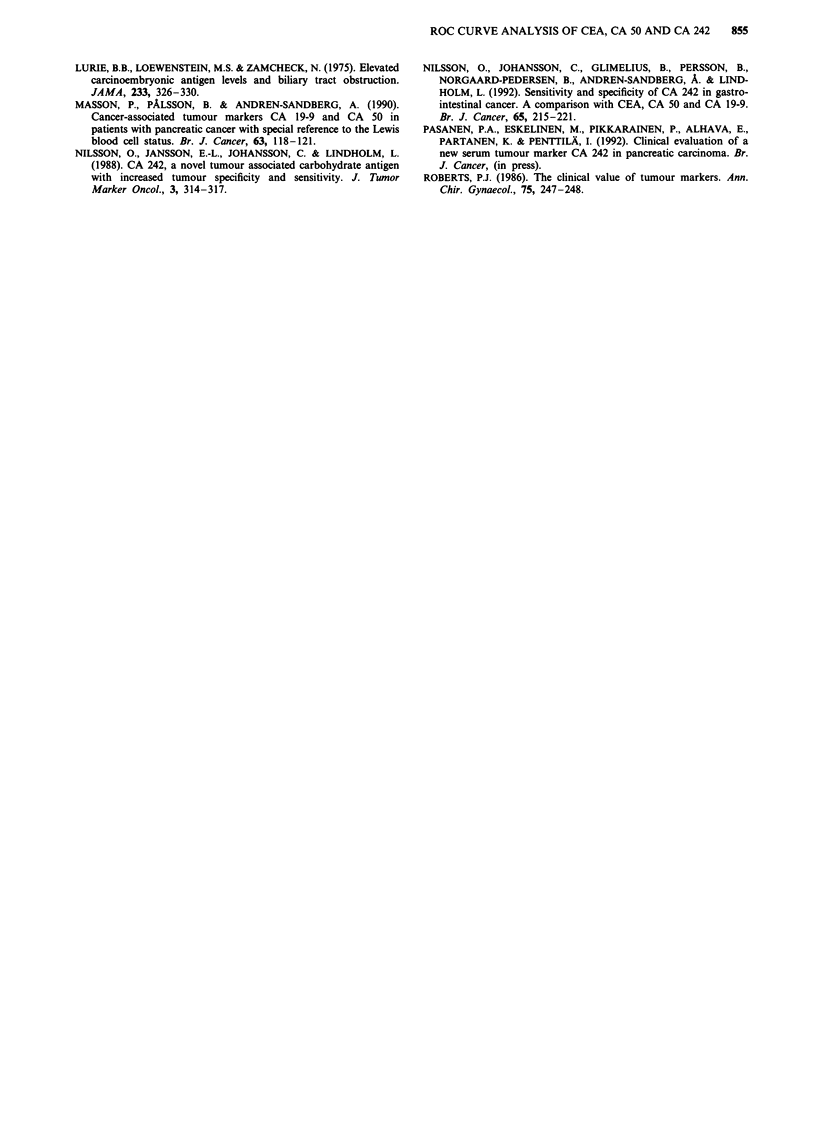# Receiver operating characteristic (ROC) curve analysis of the tumour markers CEA, CA 50 and CA 242 in pancreatic cancer; results from a prospective study.

**DOI:** 10.1038/bjc.1993.156

**Published:** 1993-04

**Authors:** P. A. Pasanen, M. Eskelinen, K. Partanen, P. Pikkarainen, I. Penttilä, E. Alhava

**Affiliations:** Department of Surgery, Kuopio University Hospital, Finland.

## Abstract

The serum values of the tumour markers carcinoembryonic antigen (CEA), cancer-associated carboanhydrate antigens CA 50 and CA 242 were evaluated in 193 patients with hepatopancreato-biliary diseases by receiver operating characteristic (ROC) curve analysis in order to compare their diagnostic accuracy in pancreatic cancer (n = 26), and to define optimal cut-off levels for the serum values of these tumour markers in the diagnosis of pancreatic cancer. The ROC analysis showed that all marker tests are considerably sensitive (77-81%) at the specificity level of 80%. The CA 242 test was more sensitive than CEA and CA 50 at high specificity levels (> 0.90) but slightly less sensitive at low specificity levels (< 0.60). The CEA test and CA 50 test performed equally well at high and low specificity levels. According to this study, it would seem optimal to use the cut-off level of 4.1 ng ml-1 for CEA, and the level of 137 U ml-1 for CA 50, since they gave a sensitivity of 77% at the specificity levels of 83% and 84%, respectively. For CA 242 the optimal cut-off level was 21 U ml-1, which gave a sensitivity and specificity of 81%. In conclusion, the results of ROC curve analysis suggest that the CA 242 test has an advantage over CEA and CA 50 because of its higher specificity in pancreatic cancer. In addition, it would seem reasonable to use higher cut-off values than what has been recommended for CEA and CA 50 in the diagnosis of pancreatic cancer, but for CA 242 the recommended cut-off level of 20 U ml-1 seems appropriate.


Br. J. Cancer (1993), 67, 852-855 © Macmillan Press Ltd., 1993

### Receiver operating characteristic (ROC) curve analysis of the tumour markers CEA, CA 50 and CA 242 in pancreatic cancer; results from a prospective study

P.A. Pasanen[1], M. Eskelinen[1], K. Partanen[2], P. Pikkarainen[3], I. Penttilä[4] & E. Alhava[1]

[1]Department of Surgery, [2]Department of Clinical Radiology, [3]Department of Medicine, [4]Department of Clinical Chemistry, Kuopio University Hospital, 70211 Kuopio, Finland.

**Summary** The serum values of the tumour markers carcinoembryonic antigen (CEA), cancer-associated carboanhydrate antigens CA 50 and CA 242 were evaluated in 193 patients with hepatopancreato-biliary diseases by receiver operating characteristic (ROC) curve analysis in order to compare their diagnostic accuracy in pancreatic cancer ($n = 26$), and to define optimal cut-off levels for the serum values of these tumour markers in the diagnosis of pancreatic cancer. The ROC analysis showed that all marker tests are considerably sensitive (77–81%) at the specificity level of 80%. The CA 242 test was more sensitive than CEA and CA 50 at high specificity levels ($>0.90$) but slightly less sensitive at low specificity levels ($<0.60$). The CEA test and CA 50 test performed equally well at high and low specificity levels. According to this study, it would seem optimal to use the cut-off level of 4.1 ng $ml^{-1}$ for CEA, and the level of 137 U $ml^{-1}$ for CA 50, since they gave a sensitivity of 77% at the specificity levels of 83% and 84%, respectively. For CA 242 the optimal cut-off level was 21 U $ml^{-1}$, which gave a sensitivity and specificity of 81%. In conclusion, the results of ROC curve analysis suggest that the CA 242 test has an advantage over CEA and CA 50 because of its higher specificity in pancreatic cancer. In addition, it would seem reasonable to use higher cut-off values than what has been recommended for CEA and CA 50 in the diagnosis of pancreatic cancer, but for CA 242 the recommended cut-off level of 20 U $ml^{-1}$ seems appropriate.

Evaluation of the results of various tumour marker studies is often problematic because of variance in the patient populations and in the cut-off values used in these studies. In many studies the sera of heterogenous populations (healthy blood donors, laboratory staff, patients with various types of cancer) have been used as reference patient material. This seems, however, unsatisfactory unless blind screening methods for the whole population are being sought. The diagnostic accuracy and cut-off values of the tumour marker tests should be tested in a relevant patient population (Roberts, 1986). In practice – and not least for reasons of economy – we should be able to focus these tests on patient groups with a high risk of certain types of cancer. The use of receiver operating characteristic (ROC) curve analysis has gained increasing popularity among clinical investigators (Feinstein, 1985). It makes possible comparisons of the sensitivity and specificity of different tests independently of the relative incidences of the diseases and of the cut-off level. This prospective series of 193 consecutive patients with jaundice and/or cholestasis ($n = 133$), and with a suspicion of chronic pancreatitis or pancreatic tumour ($n = 60$) offers a good opportunity for such an analysis with regard to the diagnosis of pancreatic cancer. We measured the serum values of carcinoembryonic antigen (CEA), cancer associated carboanhydrate antigens CA 50 and CA 242 in these patients, and compared them with the help of ROC curves.

### Patients

The patient population of this prospective study consisted of a consecutive series of patients admitted to or attending Kuopio University Hospital during the two-and-a-half year period from the beginning of December 1985 to the end of May 1988. The limits for inclusion to the study were defined as follows: a serum bilirubin level exceeding 40 micromoles per litre (normal value in our laboratory $\leqslant 17\ \mu mol\ l^{-1}$), and/or serum alkaline phosphatase level above 350 IU $l^{-1}$ (normal value in our laboratory $\leqslant 210$ U $l^{-1}$) in relation to serum gamma glutamyltranspeptidase level above 100 IU $l^{-1}$ (normal value in our laboratory $\leqslant 32$ U $l^{-1}$), or liver-specific alkaline phosphatase elevated. In addition to these jaundiced or cholestatic patients the following patients were included: patients with a history of two or more episodes of acute pancreatitis, patients who had continuous or recurring abdominal pain with raised serum or urine amylase levels measured at least three times, patients who had been suspected to have a pancreatic tumour or chronic pancreatitis in ultrasound or computed. tomography examination. Exclusion criteria were: age less than 15 years, pregnancy, jaundice developing in the intensive care unit, a history of recent heart surgery, insufficient cooperation, acute alcoholic pancreatitis, disseminated malignancy, parenchymal liver disease diagnosed within 2 days of admission, need for emergency surgery. One hundred and ninety-three patients were included altogether. The patients have been previously described in detail (Pasanen *et al.*, 1992). There were altogether 24 patients with the final diagnosis of carcinoma of the head of the pancreas and two patients with the diagnosis of carcinoma of the ampulla of Vater.

### Methods

Clinical assessment, laboratory tests, and imaging methods (ultrasound, computed tomography and endoscopic retrograde cholangio-pancreatography) were performed as previously decribed (Pasanen *et al.*, 1992). In addition, liver biopsy was obtained or secretin-cerulein test was performed if hepatocellular disease or chronic pancreatitis was suspected. All the patients involved in the study were scheduled for re-examination 6 months after entering the study, and the clinical data of the hospital records were reviewed retrospectively after a follow-up period of 2 years. The final diagnosis of a pancreatic cancer or cancer of the ampulla of Vater was based on histology in 16 cases, on cytology in three, on operative or endoscopic macroscopic morphologic findings in three, and on the imaging methods in four. The diagnosis of chronic pancreatitis was based on histology in seven cases, on cytology in one, on secretin-cerulein test in six, on the imaging methods in 14 and on clinical course of the disease in six.

Correspondence: P.A. Pasanen, Department of Surgery, Kuopio University Hospital, SF-70211 Kuopio, Finland.
Received 7 May 1992; and in revised form 20 November 1992.

*Assays*

Serum samples were obtained by venipuncture on the patient's admission to hospital before surgery or biopsy and all serum samples were stored frozen ( − 20°C) until analysed. Serum CEA concentrations were determined by using monoclonal antibody delayed immunofluorescence (TR-FIA, Wallac, Turku, Finland). Serum CA 50 concentrations were determined by using monoclonal antibody (C-50) delayed immunofluorescence (TR-FIA, Wallac, Turku, Finland). Serum CA 242 concentrations were determined by using a dissociation-enhanced lanthidine fluoroimmunoassay prototype kit (DELFIA; Pharmacia Diagnostics, Uppsala, Sweden) (Nilsson *et al.*, 1988). The assay was done according to the protocol recommended by the manufacturer.

The diagnostic sensitivity, specificity, positive predictive value ( = PV + ) and negative predictive value ( = PV − ) were calculated according to the following formulas:

Sensitivity = TP/(TP + FN), Specificity = TN/(TN + FP),
Positive predictive value = TP/(TP + FP),
Negative predictive value = TN/(TN + FN).
(TP = true positive, TN = true negative, FP = false positive, FN = false negative).

Receiver operating characterstic (ROC) curves were constructed by calculating the sensitivities (true positive rate) and specificities (false positive rate) of the CEA, CA 50 and CA 242 assays at several cut-off points (Feinstein, 1985). The differences in diagnostic accuracy between the marker tests were measured by McNemar's test (Armitage & Berry, 1987).

### Results

We have previously reported the results of the CEA, CA 50 and CA 242 assays in detail (Pasanen *et al.*, 1992). High serum marker levels were found in patients with pancreatic cancer and in patients with cancer of the bile ducts, but elevated values were also detected in patients with benign extrahepatic jaundice or cholestasis, and in patients with benign hepatocellular disease.

*Comparison of the tests by ROC curves*

ROC curves for CEA, CA 50 and CA 242 are presented in Figure 1. The sensitivity of each marker was determined at several specificity levels. The corresponding sensitivities and actual cut-off points producing Figure 1 are given in Table I. The results showed that CA 242 was significantly more sensitive than CEA and CA 50 at high (>0.90) specificity levels (at 95% specificity CA 242 *vs* CEA, $P = 0.035$, 95% confidence limits −0.52 and −0.02; CA 242 *vs* CA 50, $P = 0.013$, 95% confidence limits −0.54 and −0.07, McNemar's test), but slightly less sensitive at low (<0.60) specificity levels. At the specificity level of 80% the CA 242 test was still slightly more sensitive (81%) than CEA and CA 50 (both 77%).

**Figure 1** The value of serum CEA, CA 50 and CA 242 measurements in the diagnosis of pancreatic cancer ($n = 26$) among patients with benign ($n = 151$) and malignant ($n = 42$) hepatopancreato-biliary diseases as analysed with the help of ROC curves.

*Determining the cut-off levels*

When we used the recommended cut-off value of 2.5 ng ml$^{-1}$ for CEA, two pancreatic cancers remained under this level (sensitivity 92.3%), but 73 false positive diagnoses resulted (the specificity 59.2%). According to the ROC analysis the optimal cut-off level for CEA was 4.1 ng ml$^{-1}$, since up to this level the specificity improved without essentially decreasing the sensitivity, the resulting specificity being 83.2% and sensitivity 77%. Between the limits of 2.5 ng ml$^{-1}$ and 4.1 ng ml$^{-1}$ there were 45 false positive diagnoses including 16 cases of choledochal stones, 11 of acute or chronic pancreatitis, and six of benign liver disease.

When the recommended cut-off level of 17 U ml$^{-1}$ was used for CA 50, one pancreatic cancer remained undiagnosed, but 100 false positive diagnoses were made (sensitivity 96.1%, specificity 58.0%). In the ROC analysis the optimal cut-off level was 137 U ml$^{-1}$, which gave a specificity of 83.8% at the sensitivity level of 77%. Between the limits of 17 U ml$^{-1}$ and 137 U ml$^{-1}$ there were 40 false positive diagnoses which comprised 22 cases of choledochal stones, 15 of acute or chronic pancreatitis, and 11 of benign liver disease.

When we applied the recommended cut-off level of 20 U ml$^{-1}$ for CA 242, its sensitivity was 81% and specificity 79%. The sensitivity did not essentially improve even at the specificity level of 60% (Figure 1). In the ROC analysis, therefore, the optimal cut-off level for CA 242 was set to 21 U ml$^{-1}$, which gave a sensitivity of 81% at the same level of specificity. There were three false positive diagnoses between the levels of 20 U ml$^{-1}$ and 21 U ml$^{-1}$ in this series

**Table I** The sensitivities and corresponding cut-off levels for CEA, CA 50 and CA 242 tests in the detection of pancreatic cancer at specificity levels between 60–95%

| | | *Specificity (%)* | | | | | |
|---|---|---|---|---|---|---|---|
| | | *95%* | *90%* | *85%* | *80%* | *70%* | *60%* |
| *Sensitivity(%)* | CEA | 34.6 | 57.6 | 65.3 | 76.9 | 80.7 | 92.3 |
| | Cut-off (ng ml$^{-1}$) | 7.2 | 5.2 | 4.5 | 3.6 | 3.0 | 2.6 |
| | CA 50 | 30.7 | 50.0 | 73.0 | 76.9 | 88.4 | 88.4 |
| | Cut-off (U ml$^{-1}$) | 716.2 | 271.7 | 154.1 | 120.2 | 67.9 | 41.6 |
| | CA 242 | 61.5 | 61.5 | 73.0 | 80.7 | 80.7 | 84.6 |
| | Cut-off (U ml$^{-1}$) | 62.8 | 30.5 | 23.0 | 20.5 | 13.8 | 11.1 |

(two cases of choledochal stones and one of benign liver disease).

### Discussion

The reference and the cut-off values are produced for clinicians to support clinical decision making. The selected cut-off value of a laboratory test should provide the best diagnostic performance for either ruling out or ruling in the particular disease. The ROC curve analysis is a graphic method which can be used to determine this optimal cut-off level (Feinstein, 1985). In addition, it can be used in graphic comparison of the sensitivity and specificity of different diagnostic tests. This method has been successfully applied for comparing tumour marker tests (Eskelinen *et al.*, 1989; Nilsson *et al.*, 1992; Haglund *et al.*, 1992).

During recent years various serological markers have been developed in the diagnosis of pancreatic cancer and in many studies considerably high sensitivities and specificities have been reported. Serum CEA and CA 50 are among the most intensively studied tumour markers and their role as reference markers is established (Kalser *et al.*, 1978; Hansen *et al.*, 1974; Lindholm *et al.*, 1983; Jalanko *et al.*, 1985; Masson *et al.*, 1990; Haglund *et al.*, 1992). CA 242 is a novel tumour marker which has proved promising because of its high specificity (Haglund *et al.*, 1989; Kuusela *et al.*, 1991; Nilsson *et al.*, 1992; Pasanen *et al.*, 1992). Evaluation of these marker studies is often difficult because of variance and heterogenity of the reference populations, and perhaps this is one reason why the role of these markers in pancreatic cancer is not yet clearly established. The patient population of this prospective study consists of a consecutive series of patients with jaundice and/or cholestasis ($n = 133$), or with suspicion of chronic pancreatitis or pancreatic tumour ($n = 60$), and therefore it can be regarded as a relevant reference population in the diagnosis of pancreatic cancer.

In the ROC analysis of the current study all marker tests reached considerably high sensitivities (77–81%) at the specificity level of 80%. The CA 242 test was significantly more sensitive than CEA and CA 50 at high specificity levels ($>0.90$), but slightly less sensitive at low specificity levels ($<0.60$). The CEA test and CA 50 test performed equally well at high and low specificity levels (Figure 1). This analysis shows that the CA 242 test has a clear advantage over CEA and CA 50 because of its higher specificity, confirming thus the results of previous studies (Haglund *et al.*, 1989; Kuusela *et al.*, 1991; Nilsson *et al.*, 1992; Pasanen *et al.*, 1992).

The cut-off values recommended by the manufacturers for CEA (2.5 ng $ml^{-1}$) for CA 50 (17 U $ml^{-1}$), and for CA 242 (20 U $ml^{-1}$) are based on healthy blood donors. When we used these levels for our patients, very high (92–96%) sensitivities were reached for CEA and CA 50, but the specificities remained low ($<60\%$). The sensitivity of CA 242 was inferior (61.5%) to that of CEA or CA 50, but its specificity was clearly higher (79%). The ROC analysis of this study suggests that higher cut-off values for CEA and CA 50 should be used in order to optimise their use in the diagnosis of pancreatic cancer. Especially the specificity of these tests seems unacceptably low in the patients with jaundice and/or cholestasis. It has been shown in many studies that elevated CEA and CA 50 levels are seen in hepatocellular diseases and in benign biliary obstructions (Begent *et al.*, 1984; Kalser *et al.*, 1978; Lurie *et al.*,1975; Carr-Locke *et al.*, 1980; Haglund *et al.*, 1987). According to this study, it would seem optimal to use a cut-off level for CEA almost two times higher (4.1 ng $ml^{-1}$) than what has been recommended, and an almost ten times higher level for CA 50 (137 U $ml^{-1}$), since they gave a sensitivity of 77% at the specificity levels of 83% and 84%, respectively. For CA 242 the optimal cut-off level would be 21 U $ml^{-1}$, giving the sensitivity and specificity of 81%.

In conclusion, the ROC curve analysis of this study shows that the CA 242 test has an advantage over CEA and CA 50 because of its higher specificity in the diagnosis of pancreatic cancer. In addition, it would seem reasonable to use clearly higher cut-off values than what has been recommended for CEA and CA 50 in the diagnosis of pancreatic cancer, but for CA 242 the recommended value of 20 U $ml^{-1}$ seems appropriate. We propose that the ROC curve analysis should always be performed when a new tumour marker test is introduced or when different tests are compared, and special emphasis should be put on the relevance of the study population.

The authors wish to thank Mr Antero Julkunen, B.Sc., and Miss Raija Voutilainen, B.Sc., for their assistance in the assay procedure. Special thanks go to Pharmacia Diagnostics, Uppsala, Sweden, for providing us with the CA 242 and CA 50 kits for this study.

### References

ARMITAGE, P. & BERRY, G. (1987). *Statistical Methods in Medical Research*. pp. 120–123. Blackwell Scientific Publications: Oxford.

BEGENT, R.H.J. (1984). The value of carcinoembryonic antigen measurement in clinical practice. *Ann. Clin. Biochem.*, **21**, 231–238.

CARR-LOCKE, D.L. (1980). Serum and pancreatic juice carcinoembryonic antigen in pancreatic and biliary disease. *Gut*, **221**, 656–661.

ESKELINEN, M., TIKANOJA, S. & COLLAN, Y. (1989). Clinical evaluation of tumor marker CA 50 in breast cancer diagnostics. *Surg. Res. Comm.*, **6**, 107–113.

FEINSTEIN, A.R. (1985). *Clinical Epidemiology. The Architecture of Clinical Research*. W.B. Saunders: Philadelphia.

HAGLUND, C., KUUSELA, P., JALANKO, H. & ROBERTS, P.J. (1987). Serum CA 50 as a tumor marker in pancreatic cancer: a comparison with CA 19-9. *Int. J. Cancer*, **39**, 477–481.

HAGLUND, C., LINDGREN, J., ROBERTS, P., KUUSELA, P. & NORDLING, S. (1989). Tissue expression of the tumour associated antigen CA 242 in benign and malignant pancreatic lesions. A comparison with CA 50 and CA 19-9. *Br. J. Cancer*, **60**, 845–851.

HAGLUND, C., ROBERTS, P.J., JALANKO, H. & KUUSELA, P. (1992). Tumour markers CA 19-9 and CA 50 in digestive tract malignancies. *Scand. J. Gastroenterol.*, **27**, 169–174.

HANSEN, H.J., SNYDER, J.J., MILLER, E., VANDEVOORDE, J.P., MILLER, O.N., HINES, L.R. & BURNS, J.J. (1974). Carcinoembryonic antigen (CEA) assay. A laboratory adjunct in the diagnosis and management of cancer. *Human Pathol.*, **5**, 139–147.

JALANKO, H., HAGLUND, C., ROBERTS, P. & KUUSELA, P. (1985). Tumor markers in gastrointestinal cancers. In *Tumour Marker Antigen*. Holmgren, J. (ed), pp. 114–122. Studentlitteratur: Lund, Sweden.

KALSER, M.H., BARKIN, J.S., REDLHAMMER, D. & HEAL, A. (1978). Circulating carcinoembryonic antigen in pancreatic carcinoma. *Cancer*, **42**, 1468–1471.

KUUSELA, P., HAGLUND, C. & ROBERTS, P.J. (1991). Comparison of a new tumour marker CA 242 with CA 19-9, CA 50 and carcinoembryonic antigen (CEA) in digestive tract diseases. *Br. J. Cancer*, **63**, 636–640.

LINDHOLM, L., HOLMGREN, J., SVENNERHOLM, L., FREDMAN, P., NILSSON, O., PERSSON, B. & MYRVOLD, H. (1983). Monoclonal antibodies against gastrointestinal tumour-associated antigens isolated as monosialogangliosides. *Int. Arch. Allergy Appl. Immunol.*, **71**, 178–181.

LINDHOLM, L., JOHANSSON, C., JANSSON, E.-L., HALLBERG, C. & NILSSON, O. (1985). An immunoradiometric assay (IRMA) for the CA-50 antigen. In *Tumour Marker Antigen*. Holmgren, J. (ed) pp. 123–133. Studenlitteratur: Lund, Sweden.

LURIE, B.B., LOEWENSTEIN, M.S. & ZAMCHECK, N. (1975). Elevated carcinoembryonic antigen levels and biliary tract obstruction. *JAMA*, **233**, 326-330.

MASSON, P., PÅLSSON, B. & ANDREN-SANDBERG, A. (1990). Cancer-associated tumour markers CA 19-9 and CA 50 in patients with pancreatic cancer with special reference to the Lewis blood cell status. *Br. J. Cancer*, **63**, 118-121.

NILSSON, O., JANSSON, E.-L., JOHANSSON, C. & LINDHOLM, L. (1988). CA 242, a novel tumour associated carbohydrate antigen with increased tumour specificity and sensitivity. *J. Tumor Marker Oncol.*, **3**, 314-317.

NILSSON, O., JOHANSSON, C., GLIMELIUS, B., PERSSON, B., NORGAARD-PEDERSEN, B., ANDREN-SANDBERG, Å. & LINDHOLM, L. (1992). Sensitivity and specificity of CA 242 in gastro-intestinal cancer. A comparison with CEA, CA 50 and CA 19-9. *Br. J. Cancer*, **65**, 215-221.

PASANEN, P.A., ESKELINEN, M., PIKKARAINEN, P., ALHAVA, E., PARTANEN, K. & PENTTILÄ, I. (1992). Clinical evaluation of a new serum tumour marker CA 242 in pancreatic carcinoma. *Br. J. Cancer*, (in press).

ROBERTS, P.J. (1986). The clinical value of tumour markers. *Ann. Chir. Gynaecol.*, **75**, 247-248.